# CD7-directed CAR T-cell therapy: a potential immunotherapy strategy for relapsed/refractory acute myeloid leukemia

**DOI:** 10.1186/s40164-022-00318-6

**Published:** 2022-09-29

**Authors:** Xuanqi Cao, Haiping Dai, Qingya Cui, Zheng Li, Wenhong Shen, Jinlan Pan, Hongjie Shen, Qinfen Ma, Mengyun Li, Sifan Chen, Juncheng Chen, Xiaming Zhu, Huimin Meng, Lin Yang, Depei Wu, Xiaowen Tang

**Affiliations:** 1 National Clinical Research Center for Hematologic Diseases, Jiangsu Institute of Hematology, The First Affiliated Hospital of Soochow University, Suzhou, 215006 China; 2 Institute of Blood and Marrow Transplantation, Collaborative Innovation Center of Hematology, Soochow University, Suzhou, 215123 China; 3 PersonGen BioTherapeutics (Suzhou) Co., Ltd., Suzhou, 215123 China; 4 The Cyrus Tang Hematology Center, Soochow University, Suzhou, 215123 China; 5 Department of Hematology, The First Affiliated Hospital of Soochow University, Jiangsu Institute of Hematology, Suzhou, 215006 China

**Keywords:** Chimeric antigen receptor T‑cells, CD7, Acute myeloid leukemia, Relapsed/refractory

## Abstract

Relapsed/refractory acute myeloid leukemia (AML) patients generally have a dismal prognosis and the treatment remains challenging. Due to the expression of CD7 on 30% AML and not on normal myeloid and erythroid cells, CD7 is an attractive target for immunotherapy of AML. CD7-targeted CAR T-cells had demonstrated encouraging efficacy in xenograft models of AML. We report here on the use of autologous CD7 CAR T-cells in the treatment of a relapsed/refractory AML patient with complex karyotype, *TP53* deletion, *FLT3-ITD* mutation, and *SKAP2-RUNX1* fusion gene. Before the CAR T-cell therapy, the patient achieved partial remission with IA regimen and attained complete remission after reinduction therapy (decitabine and venentoclax). Relapse occurred after consolidation (CLAG regimen). Then she failed CLIA regimen combined with venetoclax and exhibited resistance to FLT3 inhibitors. Bone marrow showed 20% blasts (CD7+ 95.6%). A total dose of 5 × 10^6^/kg CD7 CAR T-cells was administered after the decitabine +FC regimen. Seventeen days after CAR T-cells infusion, she achieved morphologic leukemia-free state. The patient developed grade 3 cytokine release syndrome. No severe organ toxicity or immune effector cell-associated neurotoxicity syndrome was observed. In summary, the autologous CD7 CAR T-cell therapy could be considered a potential approach for AML with CD7 expression (NCT04762485).

*Trial registration* Clinical Trials.gov, NCT04762485. Registered on February 21, 2021, prospectively registered

**Supplementary Information:**

The online version contains supplementary material available at 10.1186/s40164-022-00318-6.

To the Editor:

Relapsed/refractory (r/r) acute myeloid leukemia (AML) patients generally have a dismal prognosis. Salvage treatments for r/r AML remain particularly challenging in those without targetable mutations or resistant to target agents. Anti CD33, CLL-1, and CD38 chimeric antigen receptor (CAR) T-cell therapy have been applied for the treatment of r/r AML [1–4], which have limitations of “on-target off-tumor” toxicity on normal hematopoietic stem cells or capillary leaking syndrome [5]. CD7 is expressed in approximately 30% AML whereas not expressed in normal myeloid and erythroid cells [6, 7]. Anti-CD7 CAR T-cells demonstrated encouraging efficacy for treating AML in xenograft models [8]. Here, we report the application of autologous CD7 CAR T-cells in an r/r AML patient with complex karyotype, *TP53* deletion, *FLT3-ITD* mutation, and *SKAP2-RUNX1* fusion gene.

The patient was a 17-year-old female, diagnosed with AML in May 2021. SNP array revealed a complex karyotype (Additional file 1: Table S1). Molecular biology analysis found *ASXL1* (VAF = 6%), *FLT3-ITD* (AR = 59.4%) gene mutation, and *TP53* deletion (proportion = 72%) (Fig. 2d, Additional file 1: Table S2). The patient achieved partial remission with “3 + 7” regimen (IA). Then reinduction therapy (decitabine and venetoclax) was initiated and complete remission (CR) was attained. Afterwards, she received consolidation with the CLAG regimen and sorafenib. Relapse occurred one month after this consolidation. A new *SKAP2-RUNX1* fusion gene was identified using targeted transcriptome RNA sequencing (Additional file 1: Table S3). Since she failed reinduction with the CLIA regimen (cladribine, idarubicin, low-dose cytarabine) combined with venetoclax, and gilteritinib [9], she was enrolled in our CD7 CAR T-cell therapy clinical trial (NCT04762485) (Additional file 1: Fig. S2) after informed consent was taken from her parents. Autologous CD7 CAR T-cells were prepared as the recent report [10] and the CD7 CAR configuration was shown in our previous work [11].

Before the CD7 CAR T-cells infusion, blasts in bone marrow (BM) were 20% (Fig. 2b). Flow cytometry analysis (FCM) demonstrated 12.9% of blasts that had the expression pattern CD34+CD117+CD13+CD33+CD7+CD38+CD45+CD10−CD19−. Of note, the CD7 expression was 95.6% (Fig. 2c). *FLT3-ITD* and *SKAP2-RUNX1* fusion gene remained positive as described in Fig. 2d. Lymphodepletion chemotherapy (decitabine 50 mg/day, day-6 to -3, fludarabine 30 mg/m^2^/day, day-5 to -3, and cyclophosphamide 300 mg/m^2^/day, day-5 to -3) was performed. Two days after the chemotherapy, autologous CD7 CAR T-cells were infused at a total dose of 5 × 10^6^/kg by dose escalation within 2 days (d0 1 × 10^6^/kg, d2 4 × 10^6^/kg) (Fig. 1a).

**Fig. 1:**
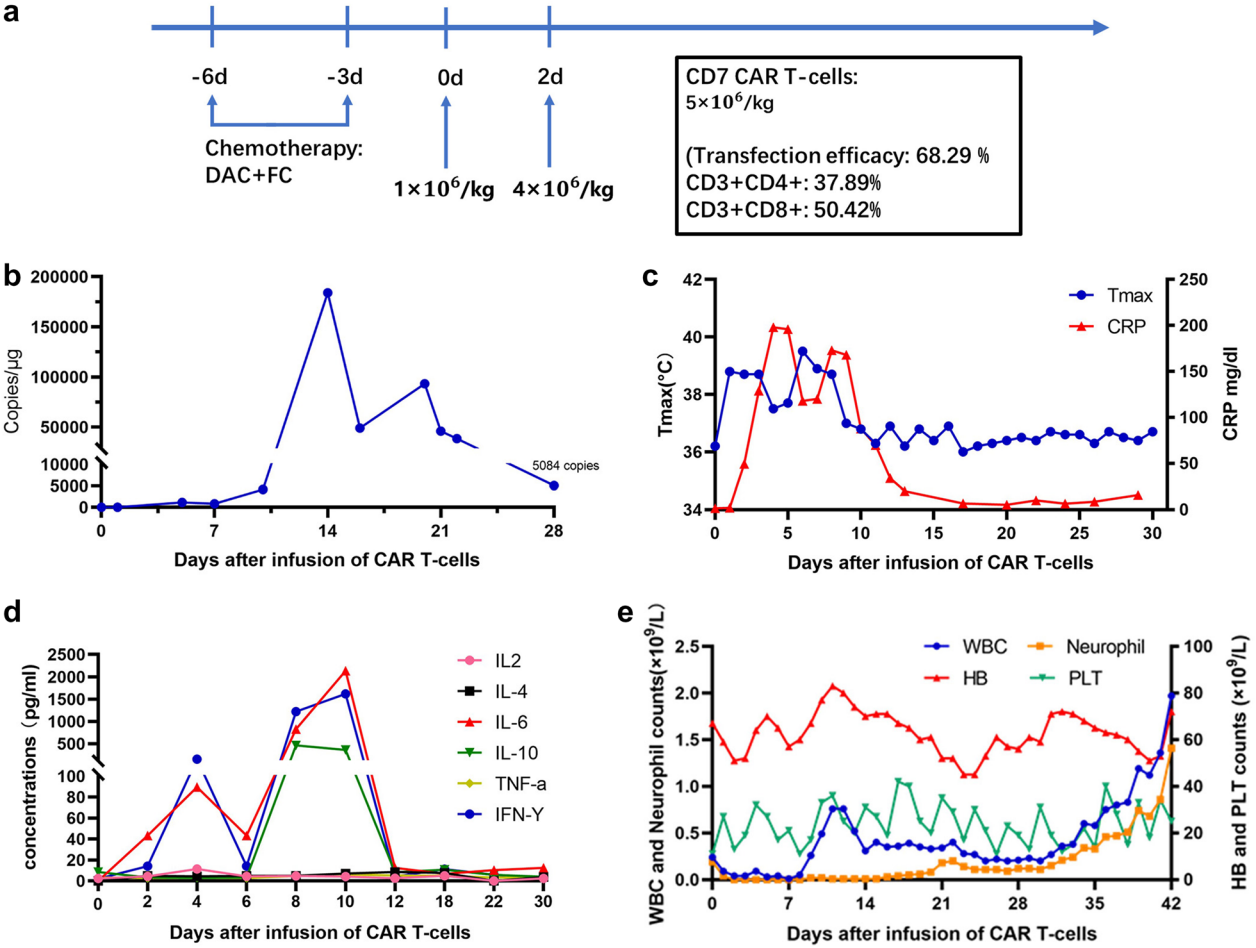
CD7 CAR T-cell therapy regime and clinical characteristic after infusion. **a** Schematic of the CD7 CAR T-cell therapy regimen, the total infusion dose of CAR T-cells was 5 × 10^6^/kg for 2 days; **b** qPCR analysis of the CAR T-cells copies in PB after the infusion. The highest level was on day 14. The patient still has 5,084 CAR-T copies/µg  by day 28; **c** Change of the temperature and CRP after CD7 CAR T-cells infusion; **d** Change of cytokines after CD7 CAR T-cells infusion; **e** Change of the blood cell counts after CD7 CAR T-cells infusion

The patient developed persistent high fever (maximum 39.4 °C, lasting for 12 days) (Fig. 1c), hypotension, grade 4 cytopenia, grade 3 liver dysfunction, and elevated serum IL-6, IL-10, and IFN-γ (Fig. 1d, Additional file 1: Fig. S3) after CAR T-cells infusion. Grade 3 cytokine release syndrome was considered [12, 13]. The toxicities were manageable with a low dose of dexamethasone, norepinephrine, and general supportive care modalities. No signs of severe infections and immune effector cell-associated neurotoxicity syndrome (ICANS) were observed. The patient’s neutropenia persisted for 38 days and the platelets were out of transfusion until 36 days after allogeneic hematopoietic stem cell transplantation (allo-HSCT) (Fig. 1e).

BM aspirates showed no blasts at 17 days after CD7 CAR T-cells infusion and minimal residual disease was 2.5 × 10^–4^ by FCM (Fig. 2a, b). Karyotype was normal and FISH analysis showed the proportion of *TP53* deletion decreased to 9%. The AR of *FLT3-ITD* mutation decreased to 5.9% and the *SKAP2-RUNX1* fusion gene decreased to 3.0%. CAR T-cells in the peripheral blood peaked at 183,945 copies/μg by qPCR on the 14th day after infusion, which were still 5,084 copies/μg on day 28 post CAR T-cell therapy (Fig. 1b). The CD7-positive T and NK cells decreased significantly as detected by FCM after CAR T-cell therapy, but CD7 negative T-cells retained the immune functions necessary for infection prevention (Fig. 2c, Additional file 1: Figs. S4, S5). Two months after the infusion, the patient underwent allo-HSCT and achieved CR without minimal residual disease (Fig. 2d).

**Fig. 2:**
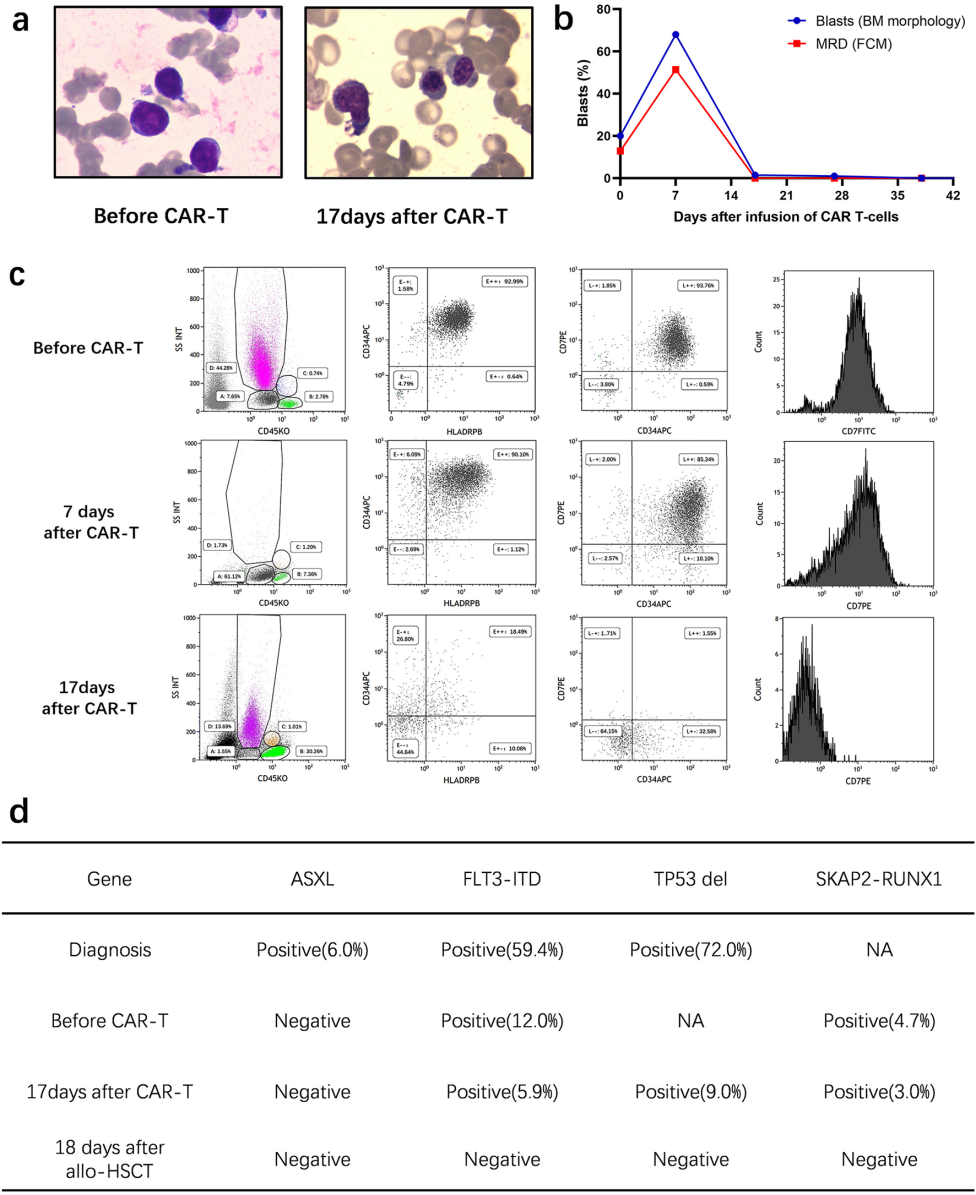
Treatment response of CD7 CAR T-cells infusion. **a** BM morphology before and after CD7 CAR T-cells infusion; **b** Change of percentage of blasts and MRD in BM after CD7 CAR T-cells infusion; **c** Flow cytometry analysis in BM before and after CD7 CAR T-cells infusion; **d** Change of molecular markers before and after CD7 CAR T-cells infusion

Overall, this patient exhibited resistance to chemotherapy, venetoclax and FLT3 inhibitors due to multiple adverse genetic aberrations (*TP53* deletion, *FLT3-ITD*, and rare *RUNX1* rearrangement). CD7 CAR T-cell therapy offered an opportunity to reduce tumor burden and bridge to allo-HSCT. Treatment-related toxicity was moderate but manageable. To our knowledge, this is the first case of r/r AML successfully treated with CD7 CAR T-cell therapy. The result suggests that CD7 CAR T-cell therapy is an encouraging approach for the treatment of CD7 positive r/r AML.

## Supplementary Information

**Additional file 1: Figure S1.** Cytotoxicity and cytokines analysis of the CD7 CAR T-cells. **Figure S2.** Diagrammatic sketch of the treatments and response. **Figure S3.** Infusion-related hepatic toxicities.** Figure S4.** Flow cytometry analysis of the fraction of T-cells and NK cells in the PB after infusion.** Figure S5. **Flow cytometry of the T-cell fractions in the PB after infusion of CART cells. **Table S1. **The result of SNP array (Cytoscan 750K/HD) at diagnosis. **Table S2. **A panel of 222 genes detected by next-generation sequencing. **Table S3.** A panel of targeted transcriptome RNA sequencing (RNA-seq).

## Data Availability

The datasets used and/or analyzed during the current study are available from the corresponding author on reasonable request.

## Abbreviations

AML: Acute myeloid leukemia
Allo-HSCT: Allogeneic hematopoietic stem cell transplantation
BM: Bone marrow
CAR: Chimeric antigen receptor
CR: Complete remission
CLAG regimen: Cladribine, cytarabine, and granulocyte colony-stimulating factor
CLIA regimen: Cladribine, idarubicin, and cytarabine
FCM: Flow cytometry
IA: Idarubicin and cytarabine
MRD: Minimal residual disease
MLFS: Morphologic leukemia-free state
qPCR: Quantitative polymerase chain reaction
r/r: Relapsed/refractory

## References

[1] Wang J, Chen S, Xiao W, Li W, Wang L, Yang S (2018). CAR-T cells targeting CLL-1 as an approach to treat acute myeloid leukemia. J Hematol Oncol.

[2] Walter RB, Appelbaum FR, Estey EH, Bernstein ID (2012). Acute myeloid leukemia stem cells and CD33-targeted immunotherapy. Blood.

[3] Wermke M, Kraus S, Ehninger A, Bargou RC, Goebeler ME, Middeke JM (2021). Proof of concept for a rapidly switchable universal CAR-T platform with UniCAR-T-CD123 in relapsed/refractory AML. Blood.

[4] Cui Q, Qian C, Xu N, Kang L, Dai H, Cui W (2021). CD38-directed CAR-T cell therapy: a novel immunotherapy strategy for relapsed acute myeloid leukemia after allogeneic hematopoietic stem cell transplantation. J Hematol Oncol.

[5] Fiorenza S, Turtle CJ (2021). CAR-T cell therapy for acute myeloid leukemia: preclinical rationale, current clinical progress, and barriers to success. BioDrugs.

[6] Chang H, Yeung J, Brandwein J, Yi QL (2007). CD7 expression predicts poor disease free survival and post-remission survival in patients with acute myeloid leukemia and normal karyotype. Leuk Res.

[7] Ogata K, Yokose N, Shioi Y, Ishida Y, Tomiyama J, Hamaguchi H (2001). Reappraisal of the clinical significance of CD7 expression in association with cytogenetics in de novo acute myeloid leukaemia. Br J Haematol.

[8] Gomes-Silva D, Atilla E, Atilla PA, Mo F, Tashiro H, Srinivasan M (2019). CD7 CAR t cells for the therapy of acute myeloid leukemia. Mol Ther.

[9] Kadia TM, Reville PK, Borthakur G, Yilmaz M, Kornblau S, Alvarado Y (2021). Venetoclax plus intensive chemotherapy with cladribine, idarubicin, and cytarabine in patients with newly diagnosed acute myeloid leukaemia or high-risk myelodysplastic syndrome: a cohort from a single-centre, single-arm, phase 2 trial. Lancet Haematol.

[10] Zhang M, Chen D, Fu X, Meng H, Nan F, Sun Z (2022). Autologous Nanobody-Derived Fratricide-Resistant CD7-CAR t-cell therapy for patients with relapsed and refractory t-cell acute lymphoblastic leukemia/lymphoma. Clin Cancer Res.

[11] Dai HP, Cui W, Cui QY, Zhu WJ, Meng HM, Zhu MQ (2022). Haploidentical CD7 CAR T-cells induced remission in a patient with TP53 mutated relapsed and refractory early T-cell precursor lymphoblastic leukemia/lymphoma. Biomark Res.

[12] Lee DW, Santomasso BD, Locke FL, Ghobadi A, Turtle CJ, Brudno JN (2019). ASTCT consensus grading for cytokine release syndrome and neurologic toxicity associated with immune effector cells. Biol Blood Marrow Transpl.

[13] Porter D, Frey N, Wood PA, Weng Y, Grupp SA (2018). Grading of cytokine release syndrome associated with the CAR T cell therapy tisagenlecleucel. J Hematol Oncol.

