# Supplementary material for: CD7-directed CAR T-cell therapy: a potential immunotherapy strategy for relapsed/refractory acute myeloid leukemia
Source: Exp Hematol Oncol. 2022 Sep 29;11:67. doi: 10.1186/s40164-022-00318-6 (PMC9523980; doi:10.1186/s40164-022-00318-6)
Supplement: Supplementary file 1 — Additional file 1: Figure S1. Cytotoxicity and cytokines analysis of the CD7 CAR T-cells. Figure S2. Diagrammatic sketch of the treatments and response. Figure S3. Infusion-related hepatic toxicities. Figure S4. Flow cytometry analysis of the fraction of T-cells and NK cells in the PB after infusion. Figure S5. Flow cytometry of the T-cell fractions in the PB after infusion of CART cells. Table S1. The result of SNP array (Cytoscan 750K/HD) at diagnosis. Table S2. A panel of 222 genes detected by next-generation sequencing. Table S3. A panel of targeted transcriptome RNA sequencing (RNA-seq). [file 40164_2022_318_MOESM1_ESM.pdf]

Additional file 1

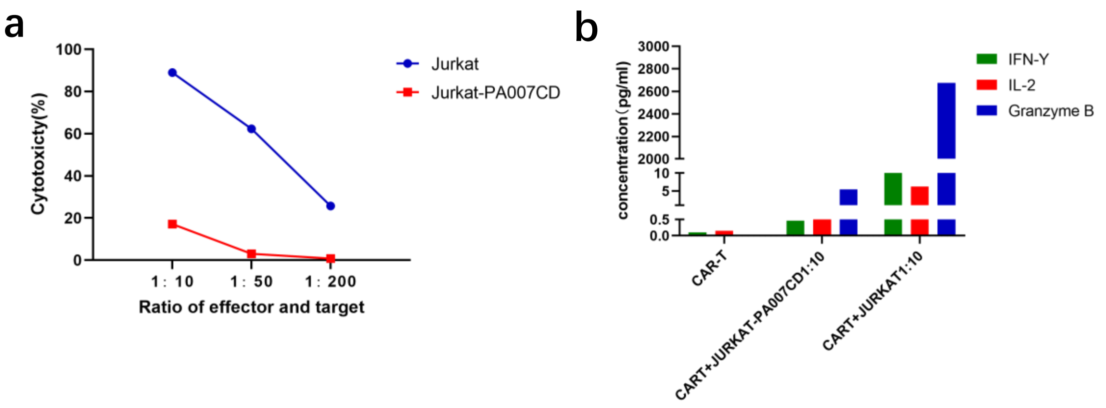

Figure S1. Cytotoxicity and cytokines analysis of CD7 CAR T-cells. **a.** Cytotoxicity analysis of CD7 CAR T-cells at different effector and target ratios; **b.** Concentrations of cytokines secreted by CAR T-cells.

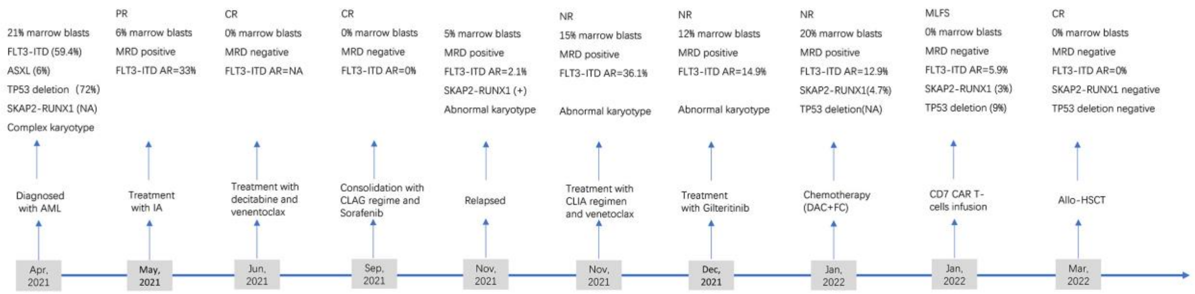

IA (May, 2021) : idarubicin 20mg/d d1-d3, cytarabine 0.185g/d d1-d7

Decitabine and venentoclax (Jun, 2021): decitabine 50mg/d d1-d3, 35mg d4, venentoclax 100mg d1, 200mg d2, 400mg/d d3-d28

CLAG (Sep, 2021): Cladribine 5 mg/m^2/d d1-d5, cytarabine 2 g/m^2/d d1-d5, G-CSF (adjust according to routine blood test)

Sorafenib (Sep, 2021): 400mg bid

CLIA regimen and venetoclax (Nov, 2021): cladribine 5 mg/m^2/d d1-d3, idarubicin 8 mg/m^2/d d1-d2, cytarabine 1 g/m^2/d d1-d3, venetoclax 400mg/d d2-d8

Gilteritinib (Dec, 2021): 600mg qd

DAC+FC (Jan, 2022): decitabine 50mg/d d-6 to d-3, fludarabine 30mg/m^2/d d-5 to d-3, cyclophosphamide 300 mg/m^2/d d-5 to d-3

Figure S2. Diagrammatic sketch of the treatments and response.

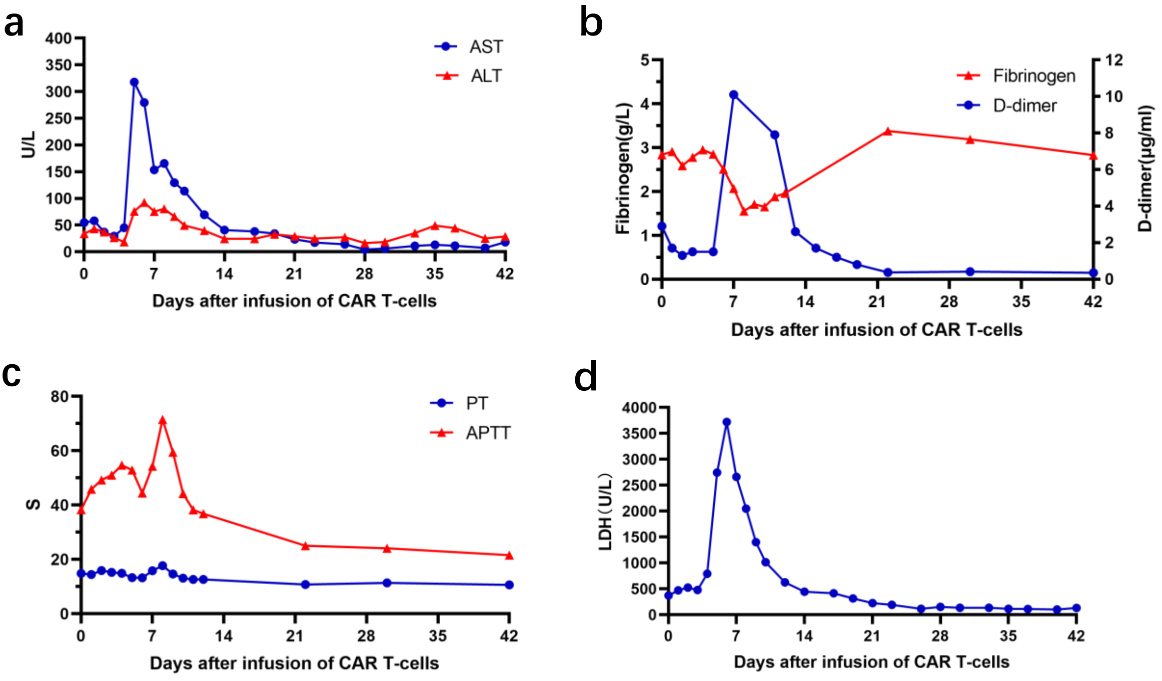

Figure S3. Infusion-related hepatic toxicities. **a.** Change of the alanine aminotransferase(ALT) and the aspartate aminotransferase (AST) after the infusion; **b.** Change of the fibrinogen and the D-dimer after the infusion; **c.** Change of the prothrombin time (PT) and the activated partial thromboplastin time （APTT） after the infusion; **d.** Change of the lactate dehydrogenase(LDH) after the infusion.

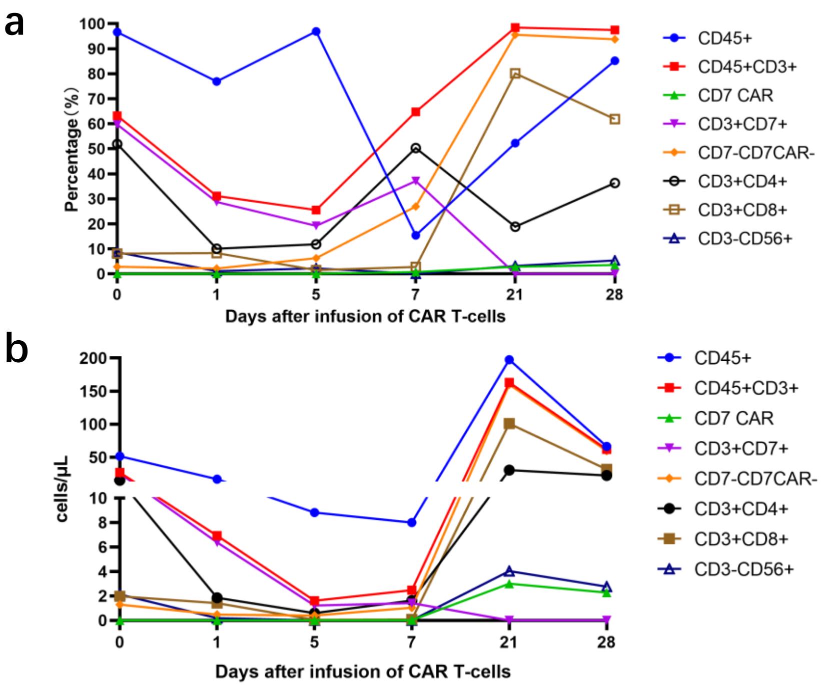

Figure S4. Flowcytometry analysis of the fraction of T-cells and NK cells in PB. **a.** The relative CAR T-cell copies, the fraction of T-cells and NK cells in PB after infusion; **b.** The absolute CAR T-cell copies, the fraction of T-cells and NK cells in PB after infusion.

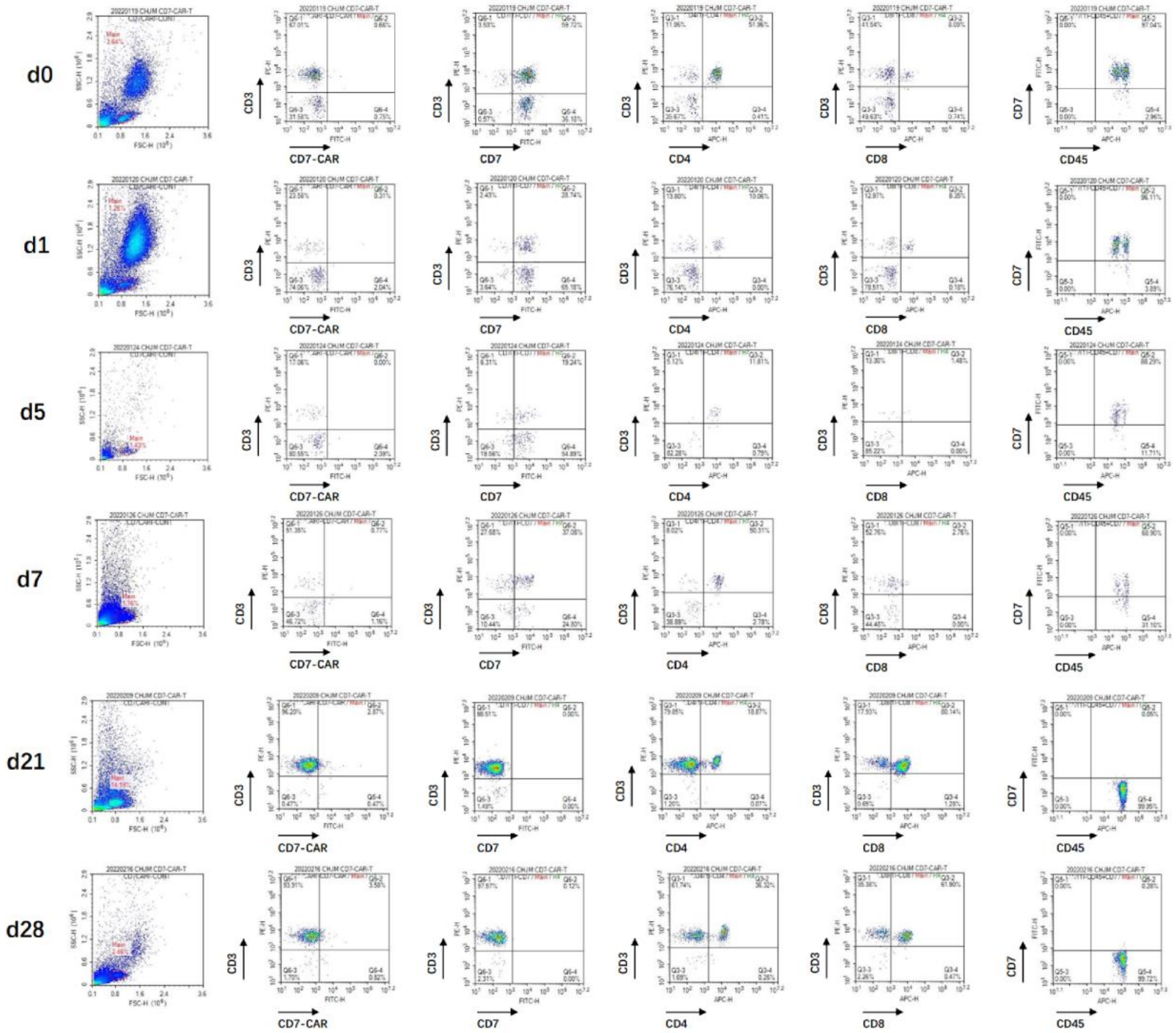

Figure S5. T-cell fractions in the PB after infusion with flowcytometry. The result of day 21 showed that the CD7 expression of T-cells had turned negative.

Chromo-some	Common disease-related genes	The location of the variant band on the chromosome (GRCh37/hg19)	type of mutation	mutation frequency (Mb)	Affected gene (quantity)
1	CFHR1 CFHR2 CFHR3 ABL2				
2					
3	CPNE4 EVI1 STAG1				
4					
5					
6	ZBTB2				
7					
8	RUNX1T1				
9	NOTCH1 CDKN2A MLLT3				
10	CTNNA3 MLLT10	10q11.22(46293591_48220168)x3	Gain	1.9	29genes
11	WT1 MLL				
12	AEBP2	12q24.31(121771435_124536319)x1	Loss	2.8	66genes
13	FLT3				
14		14q32.33(106251070_107152784)x3	Gain	0.9	8genes
15	PML				
16					
17	P53	17q11.2(29025997_30341286)x1<br>17p13.3p13.1(2233823_8236821)x1	Loss	1.3<br>6.0	23genes<br>214genes including TP53
18					
19	CEBPA				
20	ASXL1				
21	RUNX1				
22					
X	PHF6 GATA1				
Y					

Table S1. The result of SNP array (Cytoscan 750K/HD) before CAR T-cells infusion. Although the result showed normal diploid karyotype, the chromosome had 4 abnormalities (>1Mb), especially TP53 deletion, which can be considered as a complex molecular karyotype.

ABL1	BRAF	CD79B	CXCR4	EZH2	IKZF3	MED12	PDGFRA	RELN	STAG2	TP63
ANKRD26	BRCA1	CDKN1A	DDX3X	FAM46C	IL7R	MEF2B	PDGFRB	RHOA	STAT1	TP73
APC	BRCA2	CDKN2A	DDX41	FAT1	IRF4	MPL	PHF6	RPS15	STAT2	TRAF3
ARID1A	BRCC3	CDKN2B	DHX15	FBXW7	JAK1	MUM1	PIGA	RUNX1	STAT3	TYK2
ARID1B	BTG1	CDKN2C	DIS3	FLT3	JAK2	MYC	PIK3CA	SAMHD1	STAT4	U2AF1
ARID2	BTK	CEBPA	DNM2	FOXO1	JAK3	MYD88	PIM1	SETBP1	STAT5A	UNC13D
ARID5B	CALR	CEBPE	DNMT3A	GATA1	KDM6A	NF1	PLCG1	SETD2	STAT5B	WT1
ASXL1	CARD11	CHD8	ECT2L	GATA2	KIT	NFKB1	PLCG2	SF3B1	STAT6	XPO1
ASXL2	CBL	CIITA	EED	GATA3	KLF2	NFKB2	PPM1D	SH2B3	SUZ12	ZAP70
ATM	CCND1	CRBN	EGFR	GNA13	KMT2A	NFKBIE	PRDM1	SMC1A	TBL1XR1	ZBTB7A
B2M	CCND2	CREBBP	EGR2	HRAS	KMT2C	NOTCH1	PTEN	SMC2	TCF3	ZMYM3
BCL2	CCND3	CRLF2	EP300	ID3	KMT2D	NOTCH2	PTPN11	SMC3	TET1	ZRSR2
BCL6	CD274	CSF1R	EPOR	IDH1	KRAS	NPM1	PTPRD	SOS1	TET2	
BCOR	CD28	CSF3R	ERBB3	IDH2	MAP2K1	NRAS	RAD21	SPEN	TNFAIP3	
BCORL1	CD58	CSMD1	ETNK1	IGLL5	MAPK1	PAX5	RAF1	SRP72	TNFRSF14	
BIRC3	CD79A	CTCF	ETV6	IKZF1	MECOM	PDCD1LG2	RB1	SRSF2	TP53	

Table S2. a panel (222 genes) of next generation sequencing.

myeloid neoplasms with eosinophilia	PDGFRA, PDGFRB, FGFR1, JAK2, ABL1, FLT3
acute myeloid leukemia	BCR, ABL1, PML, RARA, RARB, RARG, RUNX1, CBFB, KMT2A, NUP98, NUP214, GATA2, MECOM, FLT3, MLLT10, NPM1, RBM15, CBFA2T3, ETV6
acute lymphoblastic leukemia	BCR, ABL1, ETV6, RUNX1, MEF2D, ZNF384, IL3, TCF3, PBX1, ERG, DUX4, LYN, IKZF1, PAX5, TAL1, MYC
Ph like acute lymphoblastic leukemia	ABL1, ABL2, PDGFRA, PDGFRB, FGFR1, P2RY8, CRLF2, EPOR, JAK1, JAK2, JAK3, TYK2, FLT3, CSF1R, PTK2B, NTRK3

Table S3. a panel of targeted transcriptome RNA sequencing(RNA-seq).
